# The Effect of Female Sex on Short-Term Outcomes of Patients Undergoing Off-Pump *Versus* On-Pump Coronary Artery Bypass Grafting

**DOI:** 10.21470/1678-9741-2021-0301

**Published:** 2023

**Authors:** Jun Fan, Shao-ling Luo, Yi-chao Pan, Tian-yuan Wu, Yu Chen, Wei-jie Li

**Affiliations:** 1 Department of Cardiology, Guangzhou First People’s Hospital, School of Medicine, South China University of Technology, Guangzhou, People’s Republic of China.

**Keywords:** Coronary Artery Bypass, Myocardial Infarction, Gender, Heart Disease Risk Factors, Treatment Outcome

## Abstract

**Introduction:**

According to the American Heart Association guideline for coronary artery bypass grafting (CABG), female patients undergoing on-pump CABG (ONCAB) are at higher risk of short-term adverse outcomes than male patients. However, whether off-pump CABG (OPCAB) can improve the short-term outcome of female patients compared to ONCAB remains unclear.

**Methods:**

We conducted a meta-analysis to study the effect of the female sex on short-term outcomes of OPCAB vs. ONCAB. A total of 31,115 patients were enrolled in 12 studies, including 20,245 females who underwent ONCAB and 10,910 females who underwent OPCAB.

**Results:**

The in-hospital mortality in female patients who underwent OPCAB was significantly lower than in those in the ONCAB group with (2.7% vs. 3.4%; odds ratio [OR] 0.76; 95% confidence interval [CI] 0.65-0.89) and without (OR 0.68; 95% CI 0.52-0.89) adjustment for cardiovascular risk factor. The incidence of postoperative stroke in female patients who underwent OPCAB was lower than in those in the ONCAB group (1.2% vs. 2.1%; OR 0.59; 95% CI 0.48-0.73) before cardiovascular risk factor adjustment but was not significant (OR 0.87; 95% CI 0,66-1.16) after adjustment. There was no significant difference in the incidence of postoperative myocardial infarction between women who underwent OPCAB and those in the ONCAB group (1.3% vs. 2.3%; OR 0.88; 95% CI 0.54-1.43).

**Conclusion:**

In contrast to the American Heart Association CABG guideline, female patients who had OPCAB don’t have unfavorable outcomes compared with the ONCAB group.

## INTRODUCTION

Coronary artery disease (CAD) is the leading cause of death in both developed and developing countries^[1]^. The mortality and quality of life of CAD patients have been significantly improved by the effective application of primary^[2]^ and secondary prevention^[3]^. Clinical trials have shown that improving the management of hypertension^[4]^, diabetes mellitus^[5]^, and hyperlipidemia^[6]^ promoted better clinical outcomes in CAD patients. However, several risks factors affecting the outcomes of CAD patients remain unclear^[7]^.

Coronary artery bypass grafting (CABG) is a treatment strategy for coronary artery revascularization. According to the American Heart Association CABG guideline, the female sex is a risk factor for adverse outcomes^[8]^. In Kim et al.^[9]^ meta-analysis involving 23 studies, early mortality and complications were higher among females after CABG than among males. However, this conclusion was based on the studies of on-pump CABG (ONCAB) or studies not stratified based on the cardiopulmonary bypass technique used.

In the CABG Off or On Pump Revascularization Study^[10]^ (CORONARY) and the Randomized On/Off Bypass trial^[11]^ (ROOBY), there was no significant difference between off-pump CABG (OPCAB) and ONCAB in the 30-day mortality rate. In addition, there was no significant difference in the occurrence of myocardial infarction (MI), stroke, or renal failure requiring dialysis between OPCAB and ONCAB groups in the CORONARY study^[10]^. However, to this date, there are no reports concerning the influence of sex difference on the outcomes of OPCAB *vs.* ONCAB clinically.

The study by Attaran et al.^[12]^ was the first meta-analysis that compared the short-term outcomes between off-pump *vs.* on-pump revascularization among female patients. In this study, no statistically significant difference was observed in the 30-day mortality rate and other morbidity outcomes between the OPCAB and ONCAB groups, except for perioperative MI. Recently, several new studies in this field, including the propensity score matching (PSM) study^[13]^ and studies that were adjusted for cardiovascular risk factors^[13,14]^, have been published. This study aims to investigate the latest research to study the effect of the female sex on short-term outcomes in OPCAB *vs.* ONCAB patients.

## METHODS

**Fig. 1:**
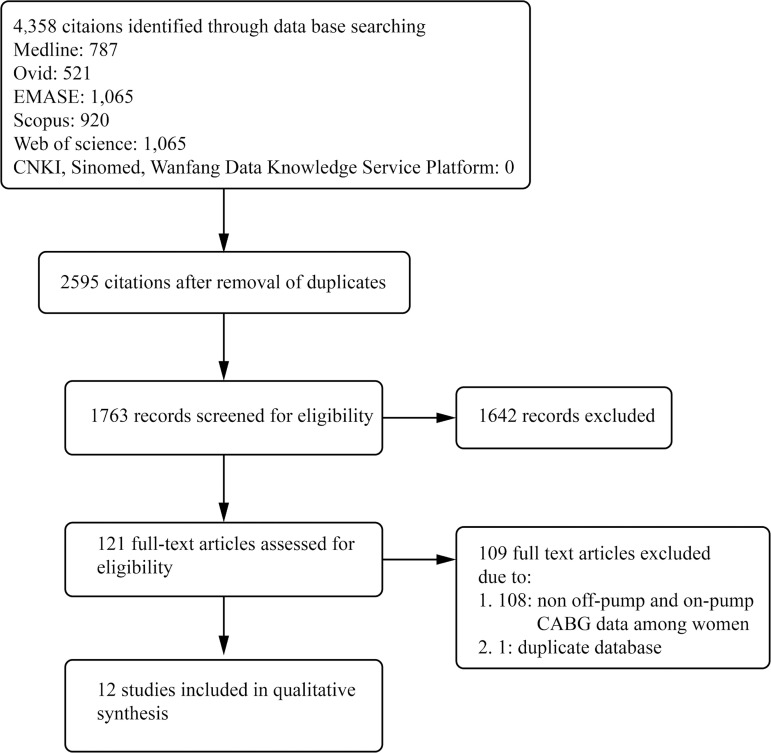
Flow diagram describing study selection in our meta-analysis. CABG=coronary artery bypass grafting; CNKI=China National Knowledge Infrastructure; SinoMed=Chinese biomedical literature service system

Since this study is a systematic review and meta-analysis based on previous articles, ethics committee approval was not required; it was conducted in accordance with the Helsinki Declaration of 1975 (revised in the year 2000). This is an observational meta-analysis that followed the guidelines for the Meta-analysis of Observational Studies in Epidemiology. This study has been registered on PROSPERO (CRD42021250888). We searched literature databases including PubMed®, Web of Science™, Embase®, Scopus™, Ovid, the China National Knowledge Infrastructure (or CNKI), the Chinese Biomedical Literature service system (or SinoMed), and the Wanfang Data Knowledge Service Platform with the keywords “coronary artery bypass”, “female”, “women”, “woman”, “gender”, and “sex”. We did not limit the start time of the studies, but we limited their end time to 2021-8-1, when retrieving the literature. After this strategy, 4,358 pieces of literature were retrieved. LSL and PYC carefully read and analyzed all the retrieved studies, and the publications were further screened according to the flow chart shown in Figure 1. Finally, 12 retrospective observational studies were included in our meta-analysis. Of the 12 studies, two were PSM studies.

We included two main types of studies in our meta-analysis: 1) studies which only included female patients grouped by OPCAB and ONCAB and 2) studies which included male and female patients undergoing CABG (OPCAB and ONCAB), but containing a clear delineation between OPCAB and ONCAB subgroups. Both types of studies must also possess documented primary and secondary endpoints.

Primary endpoints included in-hospital death, 30-day death rate after surgery, postoperative MI, and stroke. Secondary endpoints included postoperative acute renal failure (ARF), renal replacement therapy, blood transfusion, reoperation for bleeding, sternal wound infection, atrial fibrillation, and postoperative lower cardiac output.

The selected literature was not restricted by language. Abstracts, conference abstracts, and supplementary issues were also included. Patients who underwent concomitant surgical procedures such as valvular repair or replacement, correction of congenital malformation, and ascending aortic aneurysm repair, to name a few, were excluded from this study. FJ and PYC analyzed the data extracted from these studies. A consensus was reached through discussion in cases of disagreements.

### Extraction of Data

LWJ and FJ extracted data from the selected literature, including the first author’s name, the year when the study was published, the type of research, and the country where the study was conducted. General characteristics such as age, race, body mass index, and smoking status were recorded. Preoperative diseases including hypertension, diabetes, hyperlipidemia, heart failure, stroke, and peripheral vascular disease were included. Patients’ echocardiographic measurement parameters, such as left ventricular ejection fraction, were also collected. Primary and secondary endpoints were collected for investigation. The quality of the studies was evaluated according to the Newcastle-Ottawa Scale (or NOS).

### Statistical Analysis

RevMan 5.4 (Nordic Cochrane Center) statistical software was employed for meta-analysis. A *P*-value of < 0.05 was considered statistically significant. Publication bias was assessed using visual inspection of funnel plots. All included studies were retrospective in nature. A random-effects model was adopted in this study to avoid the impact of inter-study heterogeneity on the results.

## RESULTS

### Literature Retrieval

We searched the literature database as abovementioned; 4,358 scientific works were retrieved after preliminary screening. We then further screened the literature according to the strategy in Figure 1. Ultimately, 12 studies were included in our meta-analysis. All studies were retrospective observational studies. Primary and secondary endpoints were extracted for analysis.

### Characteristics of the Included Studies

**Fig. 2:**
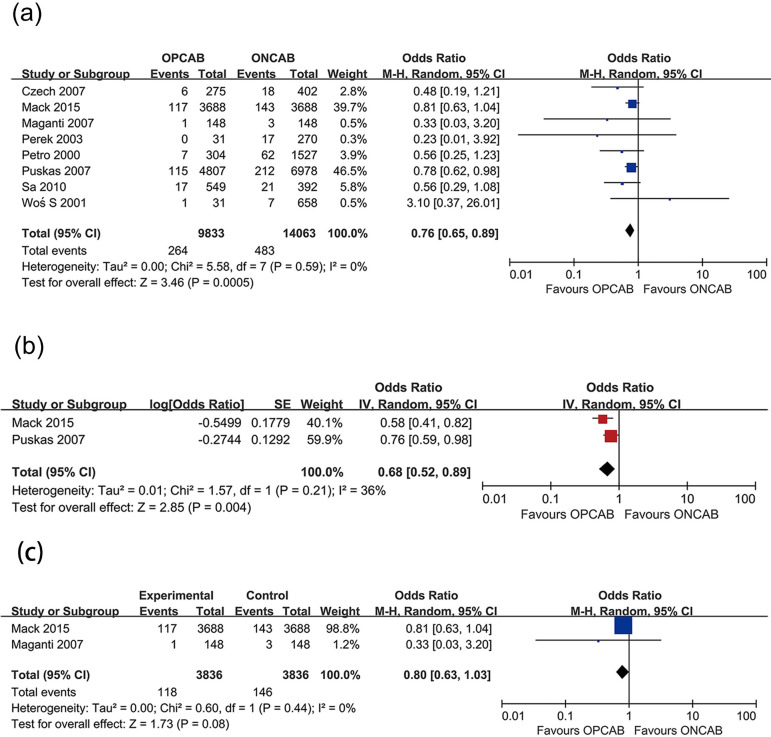
Forest plots demonstrating in-hospital mortality of off-pump coronary artery bypass grafting (OPCAB) vs. on-pump coronary artery bypass grafting (ONCAB) for (a) original data without adjustment, (b) in-hospital mortality with cardiovascular risk factor adjustment, (c) in-hospital mortality of propensity score matching studies. Chi=Chi-squared; CI=confidence interval; df=degree of freedom; IV=inverse variance; M-H=Mantel-Haenszel; SE=standard error; Tau=Tau-squared

**Fig. 3:**
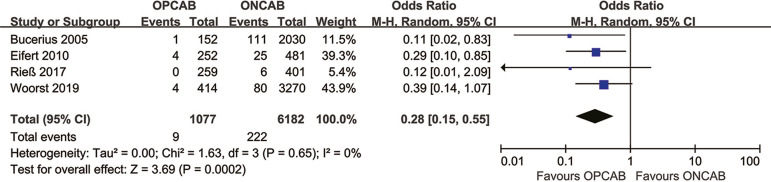
Forest plot demonstrating the 30-day hospital mortality rate of off-pump coronary artery bypass grafting (OPCAB) vs. on-pump coronary artery bypass grafting (ONCAB). Chi=Chi-squared; CI=confidence interval; df=degree of freedom; M-H=Mantel-Haenszel; Tau=Tau-squared

**Fig. 4:**
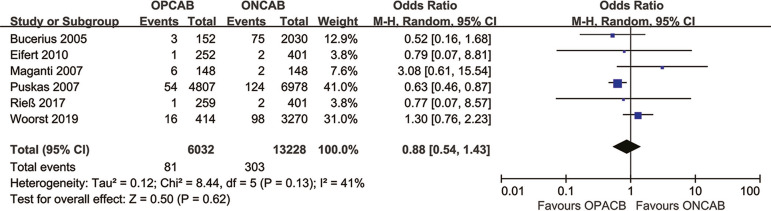
Forest plot demonstrating postoperative myocardial infarction incidence of off-pump coronary artery bypass grafting (OPCAB) vs. on-pump coronary artery bypass grafting (ONCAB). Chi=Chi-squared; CI=confidence interval; df=degree of freedom; M-H=Mantel-Haenszel; Tau=Tau-squared

**Fig. 5:**
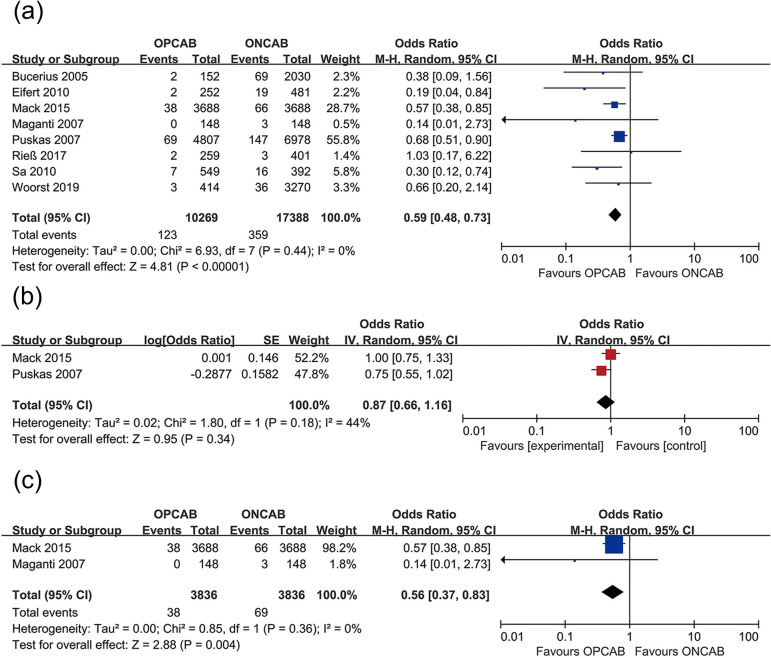
Forest plots demonstrating postoperative stroke incidence of off-pump coronary artery bypass grafting (OPCAB) vs. on-pump coronary artery bypass grafting (ONCAB) for (a) original data without adjustment, (b) stroke with cardiovascular risk factor adjustment, (c) stroke of propensity score matching studies. Chi=Chi-squared; CI=confidence interval; df=degree of freedom; IV=inverse variance; M-H=Maentel-Haenszel; SE=standard error; Tau=Tau-squared

**Supplementary Fig. 1:**
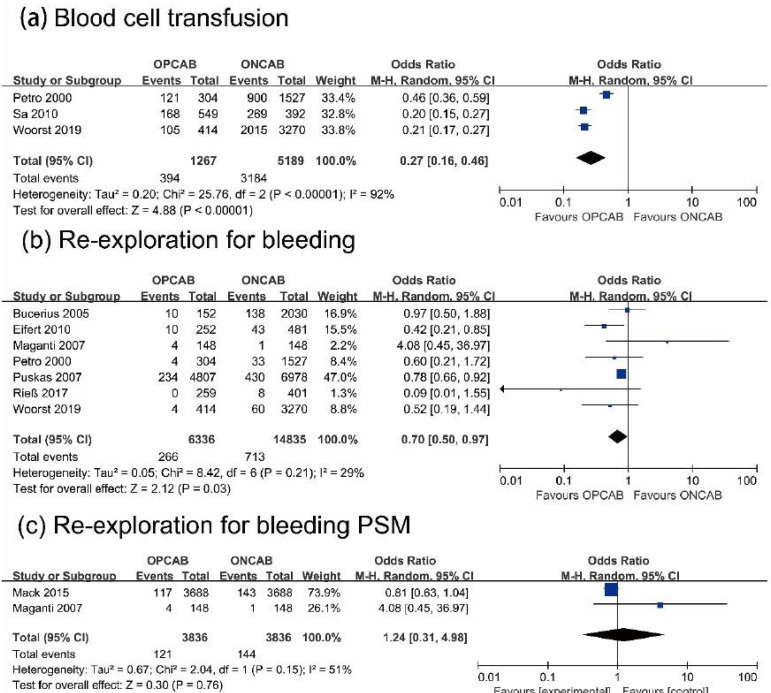
Forest plots demonstrating off-pump coronary artery bypass grafting (OPCAB) vs. on-pump coronary artery bypass grafting (ONCAB) incidence of (a) blood cell transfusion, (b) re-exploration for bleeding, (c) re-exploration for bleeding of propensity score matching (PSM) studies. Chi=Chi-squared; CI=confidence interval; df=degree of freedom; M-H=Mantel-Haenszel; Tau=Tau-squared

**Supplementary Fig. 2:**
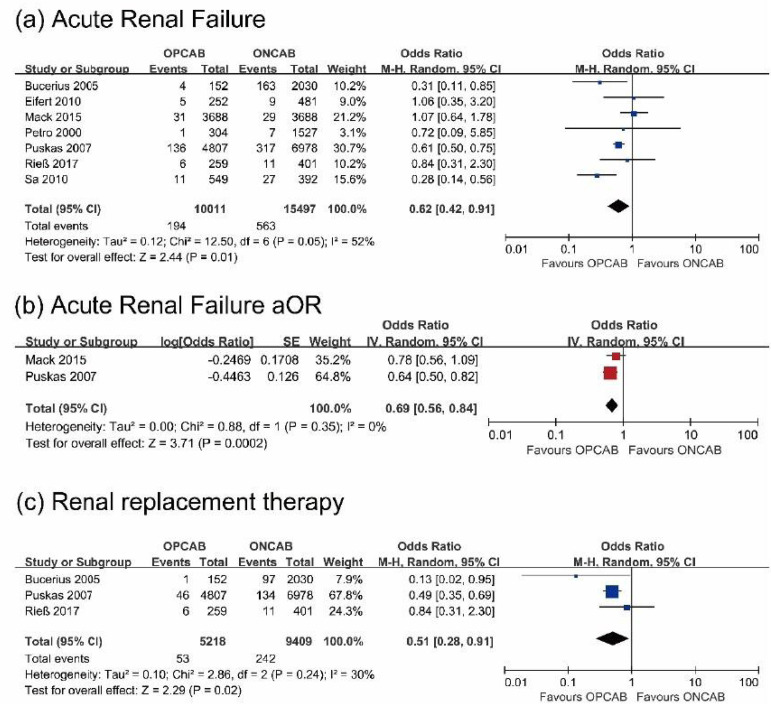
Forest plots demonstrating off-pump coronary artery bypass grafting (OPCAB) vs. on-pump coronary artery bypass grafting (ONCAB) incidence of (a) acute renal failure, (b) acute renal failure with cardiovascular risk factor adjustment, (c) renal replacement therapy. aOR=adjusted odds ratio; Chi=Chi-squared; CI=confidence interval; IV=inverse variance; df=degree of freedom; M-H=Mantel-Haenszel; SE=standard error; Tau=Tau-squared

**Supplementary Fig. 3:**
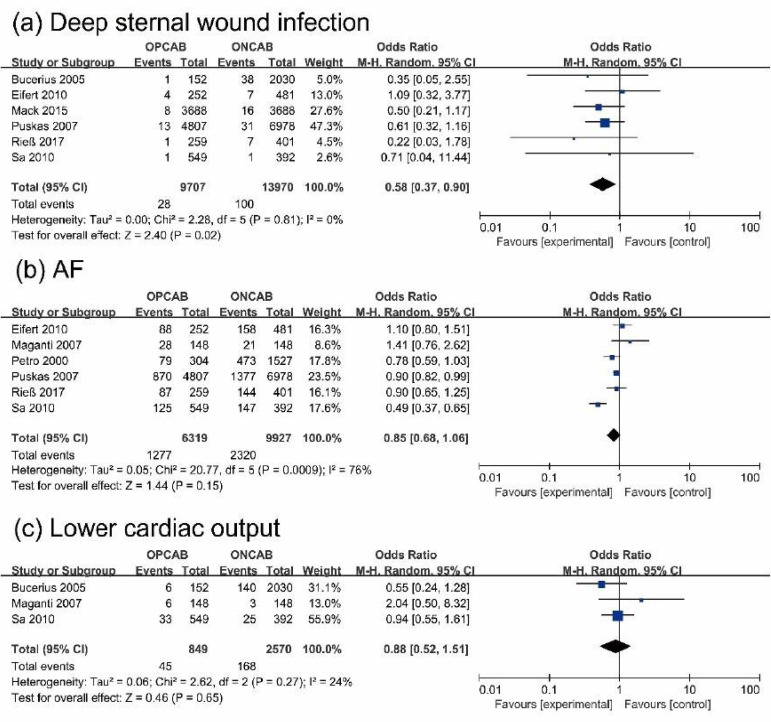
Forest plots demonstrating off-pump coronary artery bypass grafting (OPCAB) vs. on-pump coronary artery bypass grafting (ONCAB) incidence of (a) deep sternal wound infection, (b) atrial fibrillation (AF), (c) postoperative lower cardiac output. Chi=Chi-squared; CI=confidence interval; df=degree of freedom; M-H=Mantel-Haenszel; Tau=Tau-squared

**Supplementary Fig. 4:**
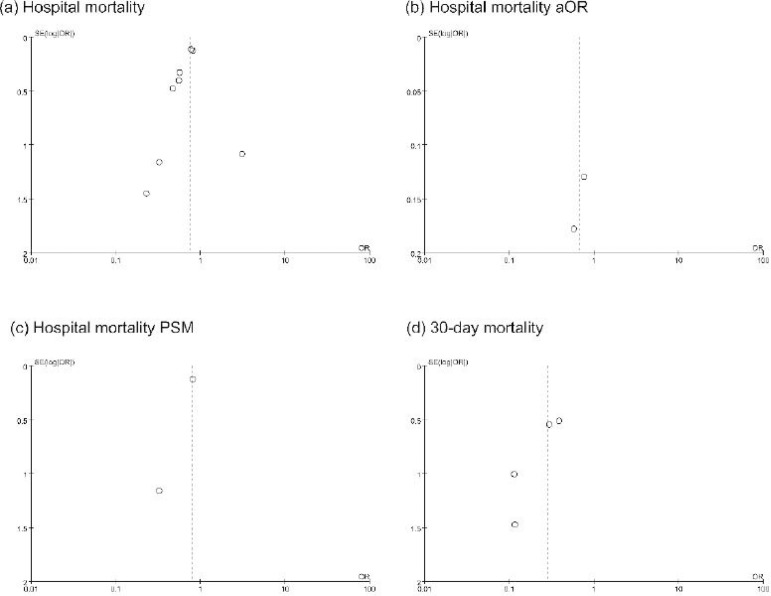
Funnel plot of off-pump coronary artery bypass grafting vs. on-pump coronary artery bypass grafting for (a) in-hospital mortality, (b) in-hospital mortality with cardiovascular risk factor adjustment, (c) in-hospital mortality of propensity score matching (PSM) studies, (d) 30-day mortality. aOR=adjusted odds ratio; OR=odds ratio; SE=standard error

**Supplementary Fig. 5:**
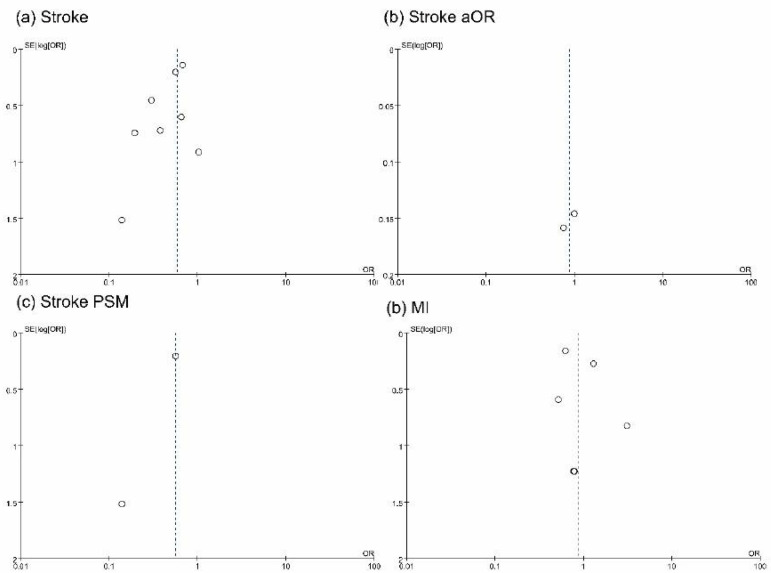
Funnel plot of off-pump coronary artery bypass grafting vs. on-pump coronary artery bypass grafting for (a) postoperative stroke incidence, (b) postoperative stroke incidence with cardiovascular risk factor adjustment, (c) postoperative stroke incidence of propensity score matching (PSM) studies, (d) postoperative myocardial infarction (MI) incidence. aOR=adjusted odds ratio; OR=odds ratio; SE=standard error

**Supplementary Fig. 6:**
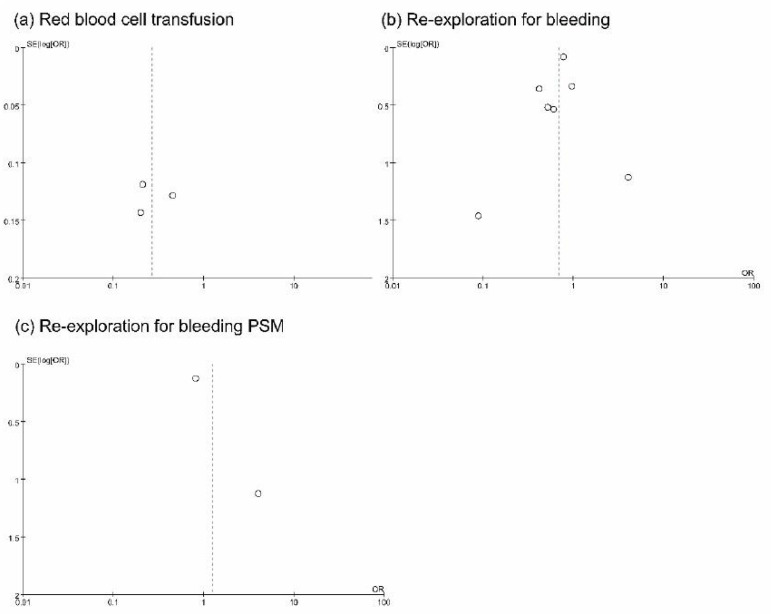
Funnel plot of off-pump coronary artery bypass grafting vs. on-pump coronary artery bypass grafting for (a) blood cell transfusion, (b) re-exploration for bleeding, (c) re-exploration for bleeding of propensity score matching (PSM) studies. OR=odds ratio; SE=standard error

**Supplementary Fig. 7:**
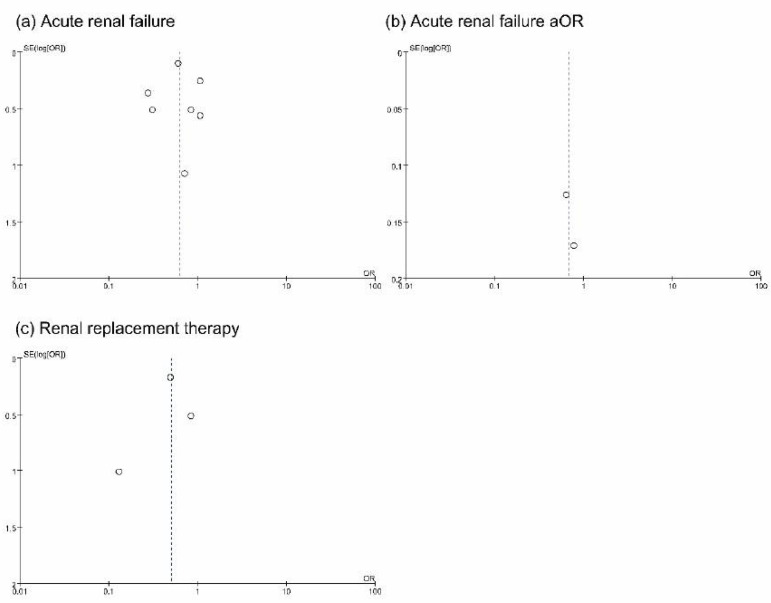
Funnel plot of off-pump coronary artery bypass grafting vs. on-pump coronary artery bypass grafting for (a) acute renal failure, (b) acute renal failure with cardiovascular risk factor adjustment, (c) renal replacement therapy. aOR=adjusted odds ratio; OR=odds ratio; SE=standard error

**Supplementary Fig. 8:**
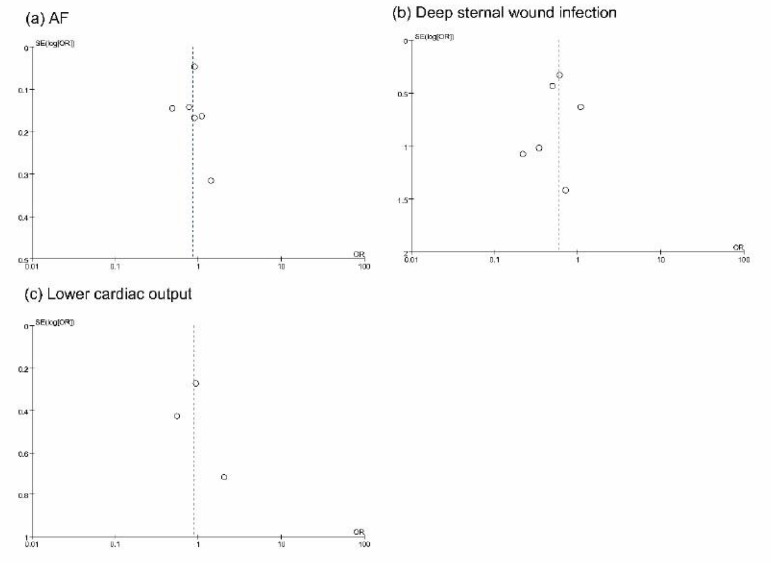
Funnel plot of off-pump coronary artery bypass grafting vs. on-pump coronary artery bypass grafting for (a) atrial fibrillation (AF), (b) deep sternal wound infection, (c) postoperative lower cardiac output. OR=odds ratio; SE=standard error

The 12 studies included were observational, three reports were from the United States of America^[13,15-18]^, and the remaining were clinical studies from Germany^[19-21]^, Netherlands^[14,22]^, Portugal^[23]^, Poland^[24-27]^, and Canada^[28,29]^. The detailed characteristics of the included patients and quality assessment are shown in Table 1. We investigated in-hospital mortality rate (Figure 2), 30-day hospital mortality rate (Figure 3), myocardial infarction incidence (Figure 4), stroke incidence (Figure 5), incidence of red blood cell transfusion and re-exploration for bleeding (Supplementary Figure 1), acute renal failure and renal replacement therapy (Supplementary Figure 2), deep wound infection (Supplementary Figure 3A), atrial fibrillation (Supplementary Figure 3B), and postoperative lower cardiac output (Supplementary Figure 3C) among female patients received ONCAB or OPCAB. A funnel plot is shown in Supplementary Figures 4-8.

**Table 1:** Characteristics of the included studies.

Source	Region	Design	Total of women, nº	OPCAB, nº	ONCAB, nº	Study quality^*^
Woorst	Netherlands	Observational	3,684	414	3,37	6
Rieß	Germany	Observational	660	259	401	4
Sá	Portugal	Observational	941	549	392	4
Eifert	Germany	Observational	733	252	481	7
Maganti	Canada	Observational	296	148	148	8
Czech	Poland	Observational	677	275	402	4
Bucerius	Canada	Observational	2,182	152	2,03	4
Mack	United States of America	Observational	7,376	3,688	3,688	4
Perek	Poland	Observational	301	31	270	4
Petro	United States of America	Observational	1,831	304	1,527	6
Puskas	United States of America	Observational	3248	1,381	1,867	6
Woś	Poland	Observational	689	31	658	4

ONCAB=on-pump coronary artery bypass grafting; OPCAB=off-pump coronary artery bypass grafting

* Newcastle-Ottawa quality assessment scale for cohort studies

### Clinical Characteristics of the Included Patients

A total of 31,115 patients were enrolled in the 12 studies, including 20,245 women who underwent ONCAB and 10,910 women who underwent OPCAB. The clinical characteristics and differences between female patients who underwent OPCAB or ONCAB, including age, hypertension, diabetes, smoking status, ejection fraction, chronic obstructive pulmonary disease, peripheral vascular disease, and previous MI, are shown in Table 2.

**Table 2:** Characteristics of study participants from the included studies.

	Age, mean, years		Diabetes, %		Hypertension, %		Dyslipidemia, %		Smoking, %		LVEF, %	
Source	OPCAB	ONCAB	OPCAB	ONCAB	OPCAB	ONCAB	OPCAB	ONCAB	OPCAB	ONCAB	OPCAB	ONCAB
Woorst	67.6 ^a^	68.8	24.4 ^a^	29.5	62.6	59.0	NR	NR	NR	NR	NR	NR
Rieß	71.6	70.4	28.2	30.2	NR	NR	NR	NR	25.1	24.7	NR	NR
Sá	68.6	69.2	45.9 ^a^	35.9	68.7 ^a^	72.1	NR	NR	14.2	12.7	NR	NR
Eifert	66.2	65.5	13.5	15	59.7	63.7	NR	NR	41.6	44.3	64.3	58.9
Maganti	65	64	41	41	70	68	74	73	NR	NR	NR	NR
Czech	NR	NR	NR	NR	NR	NR	NR	NR	NR	NR	NR	NR
Bucerius	67.9	68.1	45.4	44.6	82.2	78.3	55.3	55.9	NR	NR	NR	NR
Mack	68.6	68.9	34.6	34.2	69.5 ^a^	66.6	NR	NR	13.7	12.6	NR	NR
Perek	NR	NR	NR	NR	NR	NR	NR	NR	NR	NR	NR	NR
Petro	67	66	36 ^a^	46	NR	NR	NR	NR	NR	NR	NR	NR
Puskas	65.1	64.8	42	42	84	79	NR	NR	33	26	NR	NR
Woś	57	62	NR	NR	NR	NR	NR	NR	NR	NR	NR	NR

LVEF=left ventricular ejection fraction; NR=not reported; ONCAB=on-pump coronary artery bypass grafting; OPCAB=off-pump coronary artery bypass grafting

a Indicates a statistically significant association

### Effect of OPCAB on In-Hospital Mortality Rate Among Female Patients

We included eight studies to investigate the effect of OPCAB on in-hospital mortality in women. Female coronary heart disease patients undergoing ONCAB were the control group. A total of 23,896 women were enrolled in these eight studies, including 9,833 women who underwent OPCAB and 14,063 women who underwent ONCAB. The number of deaths in OPCAB and ONCAB patients was 264 and 483, respectively. The in-hospital mortality rate in female patients who underwent OPCAB was significantly lower than in those in the ONCAB group (2.7% *vs.* 3.4%; odds ratio [OR] 0.76; 95% confidence interval [CI] 0.65-0.89) (Figure 2A).

In two of the eight studies, OR values were corrected for cardiovascular risk factors. Consistently with the meta-analysis results of these eight studies, the mortality rate of female patients who underwent OPCAB was lower than of those in the ONCAB group (OR 0.68; 95% CI 0.52-0.89) (Figure 2B).

Among these eight studies, a PSM method was employed in two of them. A total of 3,836 female patients who underwent OPCAB and 3,836 female patients who underwent ONCAB were enrolled in these two studies. The number of deaths in OPCAB and ONCAB patients was 118 and 146, respectively. In contrast to the abovementioned results, there was no significant difference in in-hospital mortality rate between female patients who underwent OPCAB or ONCAB (3.1% *vs.* 3.8%; OR 0.80; 95% CI 0.63-1.03) (Figure 2C).

### Effect of OPCAB on 30-Day Hospital Mortality Rate Among Female Patients

We selected four studies to investigate the effect of OPCAB on the 30-day postoperative mortality rate in female patients. Female patients who underwent ONCAB were employed as the control group. A total of 7,529 women were enrolled in these four studies, including 1,077 OPCAB patients and 6,182 ONCAB patients. The female 30-day death rate of OPCAB and ONCAB were nine and 222, respectively. Patients who underwent OPCAB had a lower 30-day mortality rate than those in the ONCAB group (0.8% *vs.* 3.6%; OR 0.28; 95% CI 0.15-0.55) (Figure 3).

### Effect of OPCAB on Myocardial Infarction Incidence Among Female Patients

We included six studies to investigate the effect of OPCAB on postoperative MI in female patients. There was no significant difference in the incidence of postoperative MI in women who underwent OPCAB compared with those that underwent ONCAB (1.3% *vs*. 2.3%; OR 0.88; 95% CI 0.54-1.43). Of the 12 studies included in this study, the PSM method was employed in one, and the result of this study was consistent with previous results (Figure 4).

### Effect of OPCAB on Stroke Incidence Among Female Patients

We included eight studies to investigate the effect of OPCAB on stroke in female patients. Female patients who underwent ONCAB were used as the control group. A total of 27,657 women were enrolled in these eight studies, including 10,269 women who underwent OPCAB and 17,388 women who underwent ONCAB. The number of postoperative strokes in OPCAB and ONCAB female patients was 123 and 359, respectively. The incidence of postoperative stroke in OPCAB female patients was lower than in those in the ONCAB group (1.2% *vs.* 2.1%; OR 0.59; 95% CI 0.48 - 0.73) (Figure 5A).

Among these eight studies, two used the PSM method. Consistently with previous results, the incidence of stroke in the OPCAB group was lower than in the ONCAB group (1.0% *vs.* 1.8%; OR 0.56; 95% CI 0.37-0.83) (Figure 5B). In two of the eight studies, postoperative stroke OR values were adjusted for cardiovascular risk factors. In contrast to previous results, there was no significant difference in the incidence of postoperative stroke between the OPCAB and ONCAB groups (OR 0.87; 95% CI 0.66-1.16) (Figure 5C).

### Effect of OPCAB on the Incidence of Red Blood Cell Transfusion and Re-exploration for Bleeding

We included three studies to investigate the effect of OPCAB on blood transfusion occurrence in female patients. The incidence of blood transfusion in female patients who received OPCAB was lower than in those in the ONCAB group (31.1% *vs.* 61.4%; OR 0.27; 95% CI 0.16-0.46) (Supplementary Figure 1A).

Seven studies were included to investigate the effect of OPCAB on re-exploration for bleeding among female patients. Postoperative re-exploration bleeding was lower in female OPCAB patients than in those in the ONCAB group (4.2% *vs.* 4.8%; OR 0.70; 95% CI 0.50-0.97) (Supplementary Figure 1B). However, in the meta-analysis of PSM studies, there was no significant difference in re-exploration for bleeding incidence in female ONCAB patients compared with the control group (Supplementary Figure 1C).

### Effect of OPCAB on Acute Renal Failure and Renal Replacement Therapy in Female Patients

We included seven studies to investigate the effect of OPCAB on postoperative ARF among female patients. A total of 25,508 women were enrolled, including 10,011 women who underwent OPCAB and 15,497 women who underwent ONCAB. The incidence of ARF in OPCAB female patients was lower than in those in the ONCAB group (1.9% *vs.* 3.6%; OR 0.62; 95% CI 0.42-0.91) (Supplementary Figure 2A). Two studies that investigated OR adjusted by cardiovascular risk factors also showed a lower risk of postoperative ARF in women who underwent OPCAB (OR 0.69; 95% CI 0.56-0.84) (Supplementary Figure 2B). We also found that the incidence of female patients receiving renal replacement therapy after surgery was lower in the OPCAB than in the ONCAB group (1.02% *vs.* 2.57%; OR 0.51; 95% CI 0.28-0.91) (Supplementary Figure 2C).

### Effect of OPCAB on Deep Wound Infection in Female Patients

Six studies were included to investigate the impact of OPCAB on deep wound infection among female patients. A total of 9,707 OPCAB female patients and 13,970 ONCAB female patients were included in these studies. We found that the incidence of deep wound infection in OPCAB patients was lower than in ONCAB patients (0.3% *vs.* 0.7%; OR 0.58; 95% CI 0.37-0.90) (Supplementary Figure 3A).

### Effect of OPCAB on Atrial Fibrillation and Postoperative Lower Cardiac Output

We included six studies to investigate the effect of OPCAB on postoperative atrial fibrillation in women. A total of 6,319 female patients who underwent OPCAB and 9,927 female patients who underwent ONCAB were included in these studies. The incidence of postoperative atrial fibrillation showed no statistical difference in OPCAB patients compared with ONCAB patients (20.2% *vs.* 23.4%; OR 0.85; 95% CI 0.68-1.06) (Supplementary Figure 3B). Three studies were included to investigate the effect of OPCAB on postoperative lower cardiac output in women. The results showed no significant difference in postoperative lower cardiac output incidence in OPCAB patients compared with ONCAB patients (5.3% *vs.* 6.5%; OR 0.88; 95% CI 0.52-1.51) (Supplementary Figure 3C).

## DISCUSSION

In this study, we included 12 retrospective observational studies regarding the influence of the female sex on the short-term clinical outcomes following OPCAB and ONCAB. A total of 31,115 patients were included, which consisted of 20,245 males and 10,910 females. We observed that the incidence of adverse events in female patients who underwent OPCAB was lower or not significant, but not higher, than in those in the ONCAB group.

According to the American Heart Association guidelines for CABG, women are at a higher risk for adverse clinical outcomes, including postoperative mortality and stroke^[8]^. However, most of these studies are based on ONCAB. Previous research from Risum et al.^[29]^ confirmed that the risk of early mortality and low-output syndrome needing intra-aortic balloon support was higher in women than in men. In addition, a meta-analysis from Wognsen et al.^[30]^ found that females run an increased risk of early death and the development of postoperative complications after CABG compared with males. These results were mainly caused by the increased complexity of the procedure due to women’s smaller body surface area^[30]^.

OPCAB is performed on a beating heart without extracorporeal bypass compared with traditional extracorporeal bypass surgery. OPCAB has many advantages, such as shorter operation time, reduced hospitalization and intensive care unit length of stay, lower medical costs, and fewer surgery-related complications^[31]^.

Large randomized controlled clinical trials, including CORONARY^[10]^ and ROOBY^[11]^, found no significant difference between OPCAB and ONCAB regarding the 30-day death rate, MI, stroke, or renal failure requiring dialysis. However, these clinical studies did not investigate whether female patients who underwent OPCAB had a better short-term outcome compared to female patients who underwent ONCAB.

A meta-analysis from Attaran et al.^[12]^ investigated short-term outcomes among OPCAB *vs.* ONCAB female patients. In this study, no statistically significant difference was observed in the 30-day mortality rate and other morbidity outcomes between the OPCAB and ONCAB groups, except for perioperative MI. This study’s results are limited because both 30-day mortality and not in-hospital mortality rates were considered primary endpoints after CABG, but most of the included research investigated in-hospital mortality rates. Although 30-day mortality and in-hospital mortality rates are both short-term effects, failure to delineate them may give incorrect conclusions. Furthermore, Attaran et al.’s study did not investigate the OR adjusted by cardiovascular risk factors, leading to confounding factors affecting the results^[12]^.

In contrast to Attaran’s study, we found that the in-hospital and 30-day mortality rates in female patients who underwent OPCAB were significantly lower than in those in the ONCAB group in studies with and without cardiovascular risk factor adjustment. The in-hospital mortality rate of OPCAB female patients was not significantly different from ONCAB female patients in PSM studies. In the primary meta-analysis, the incidence of postoperative stroke in female patients who underwent OPCAB was lower than in those in the ONCAB group, while the difference in postoperative stroke between OPCAB and ONCAB in PSM studies and post-MI was insignificant. The incidence of unfavorable outcomes in female patients who underwent OPCAB was not higher than in those in the ONCAB group. In summary, the short-term clinical outcomes of women who underwent OPCAB were not worse than of those in the ONCAB group. Notably, the in-hospital mortality and postoperative 30-day mortality rates of OPCAB patients were lower than of ONCAB patients. We surmise that this may be related to the fact that OPCAB causes less trauma and minimally affects patients’ circulation compared to ONCAB.

### Limitations

Our study has the following shortcomings: 1) we had not retrieved random control studies, so the studies we included were all retrospective case-control observational studies that might attenuate our research’s strength; 2) our study did not further explore the effect of sex difference on long-term prognosis after CABG due to the lack of relevant literature.

## CONCLUSION

Compared to the American Heart Association CABG guideline, the incidence of adverse events in female patients who underwent OPCAB was lower or not significant, but not higher, than in those in the ONCAB group. Our findings should nevertheless be treated with caution due to the limitations attributed to observational studies. Randomized controlled trials are warranted to further substantiate our conclusion in the future.
